# Cervical Disc Herniation Causing Brown-Séquard's Syndrome: A Case Report and Literature Review

**DOI:** 10.1155/2011/943720

**Published:** 2012-01-22

**Authors:** Tarush Rustagi, Siddharth Badve, Hemil Maniar, Aseem N. Parekh

**Affiliations:** Department Of Orthopaedics, Topiwala National Medical College and BYL Nair Charitable Hospital, Dr. A.L. Nair Road, Mumbai Central, Mumbai 400008, India

## Abstract

Brown-Séquard's syndrome (BSS) is caused by hemisection or hemicompression of the cord leading to ipsilateral motor deficit and contralateral sensory loss. Cervical disc herniation has been reported to be a rare cause of Brown-Séquard's syndrome. We describe a rare case of multilevel cervical disc herniation presenting as BSS. The condition was confirmed by MRI scan. Cervical corpectomy, decompression, and fusion gave a satisfying result. Pertinent literature has been reviewed.

## 1. Introduction

Brown-sequard's syndrome (BSS) was first described as a clinical entity in 1849 after a traumatic hemisection of the cord from a knife [1].

BSS, which is a complete hemitransaction of cord, is unusual to find in its classic form. Mostly it is seen as an incomplete lesion with some additional features [2].

Trauma and spinal cord tumours (metastatic and intrinsic) are the most common cause of this syndrome, although other causes have been described in the literature [3–8]. Cervical disc herniation (CDH) has been reported to be a rare cause of BSS.

We report a case of Brown-sequard's syndrome caused by multiple level cervical disc herniations in a 42-year male. The literature pertinent to the case has been reviewed.

## 2. Case Report

A previously healthy 42-year-old farmer, presented with neck pain along with referred pain to the interscapular region of eight months duration. He also noticed a decrease in the right-side grip strength and slipping of footwear on the same side since five months along with tingling and burning sensation on the left side of the body since four months. There were no associated bowel/bladder or gait complaints.

**Figure 1:**
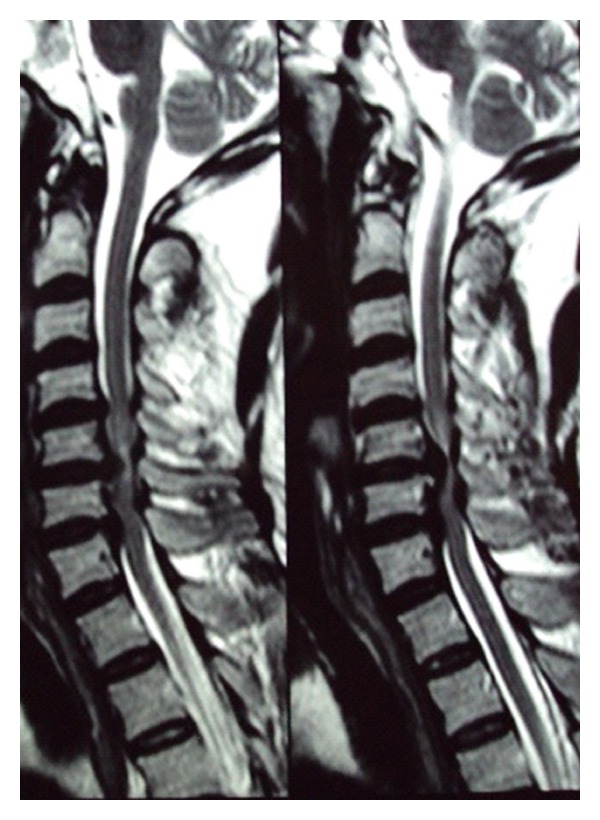
Sagittal MRI image showing C4-C5,C5-C6,C6-C7 disc herniation with caudal migration behind the vertebral body.

**Figure 2:**
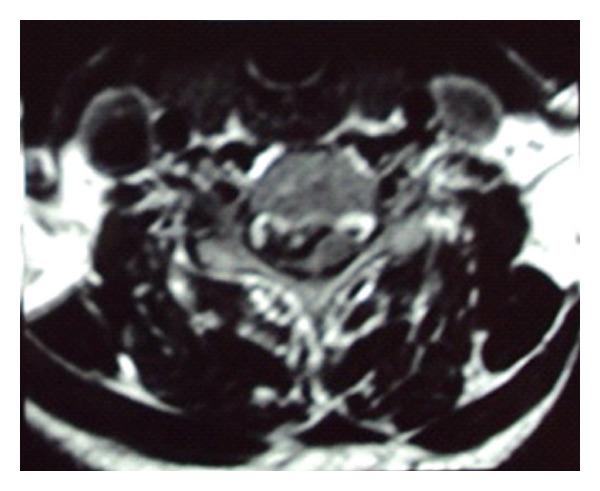
Axial MRI image at C4-C5 level showing compression of right side of cord.

**Figure 3:**
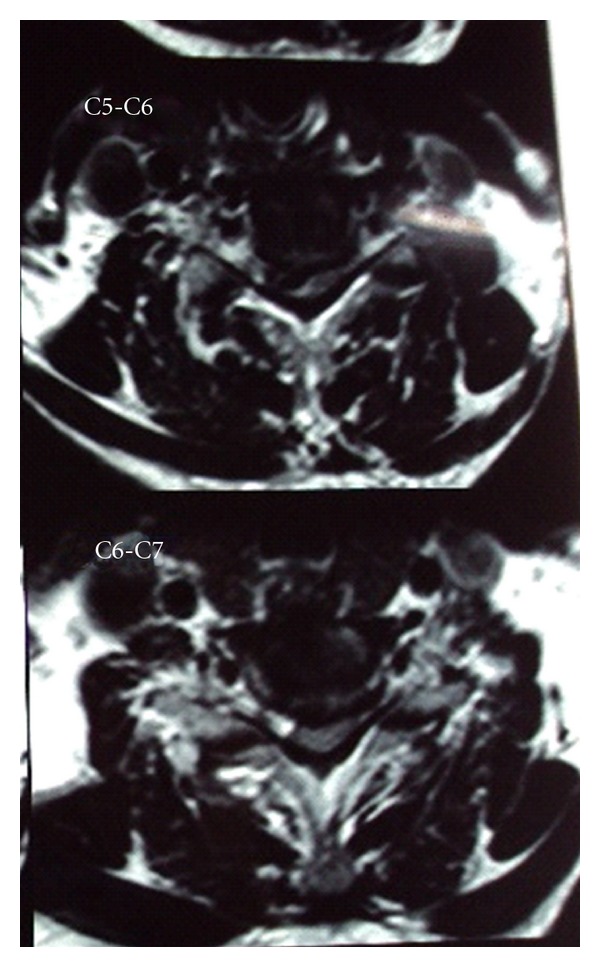
Images showing hemicompression at C5-C6,C6-C7 levels.

On physical examination there was decrease in the right side elbow extension (3/5 MRC scale) and week grip strength (20–30% of the opposite side). Also noted was a decrease in the motor power in the right lower limb at all joints (2/5 MRC). Patient had a decrease in the thermal sensitivity and response to painful stimuli on the left side below the C7 dermatome. Hyperreflexia, inverted supinator reflex, was noted on the right side with an ipsilateral positive Babinski's sign which was consistent with the diagnosis of BSS. The abdominal and the cremasteric reflex were absent on the right side. Radiographs of the cervical spine revealed evidence of spondylosis at C4–C6 vertebra. MRI scan showed cervical disc prolapsed at C4-C5, C5-C6, and C6-C7 without any evidence of myelomalacia (Figure 1). Also evident was the caudal migration of the disc behind the vertebral bodies at C5-C6 and C6-C7. The axial MRI images revealed right paracentral disc herniation causing hemicord compression (Figures 2 and 3).

**Figure 4:**
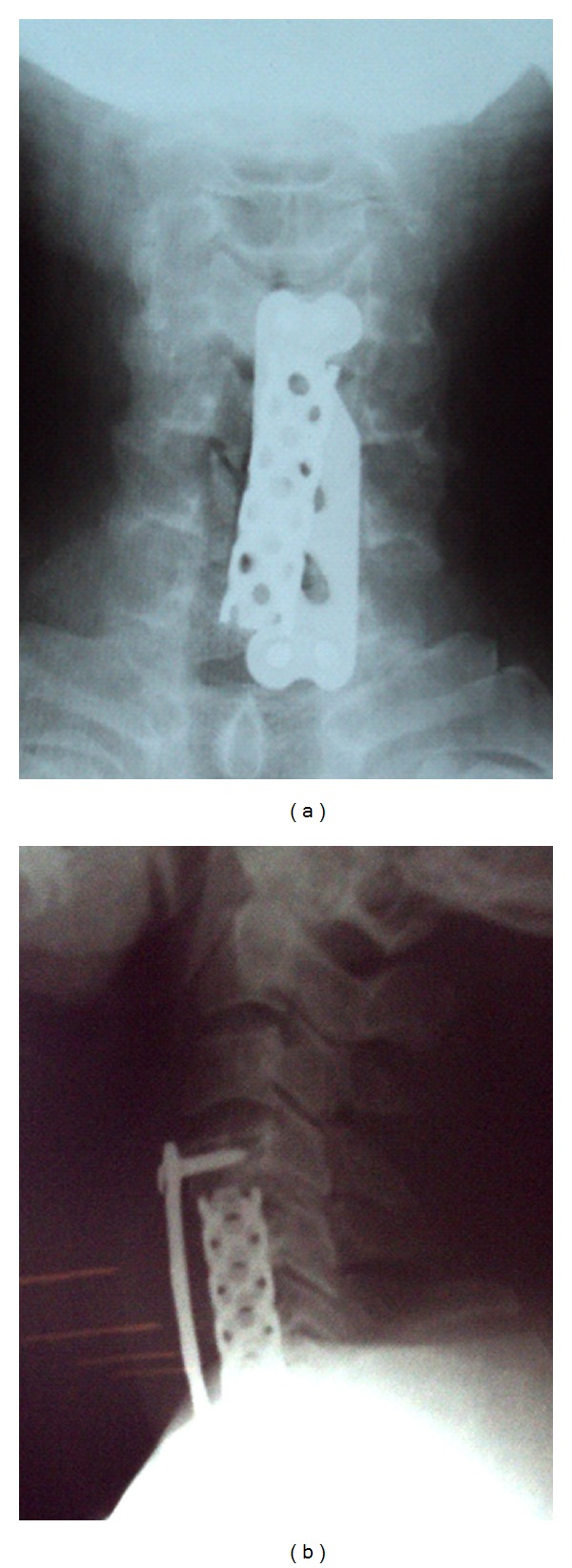
One year postoperative AP view and lateral view.

 Surgery was planned via a standard anterior approach to access the culprit discs. This included C5, C6 corpectomy, and removal of the disc material. After the decompression, interbody fusion was performed using a mesh cage with autologous bone graft which was further supported by an anterior cervical plate (Figures 4(a) and 4(b)). The patient was immobilized in a rigid cervical collar after surgery.

 Postoperatively there was significant relief in pain and the burning sensation on the left side of the body after 72 hours. There was a slow, but progressive recovery in the motor strength. At one year followup, the patient had regained grade 4/5 power in the upper limb with grip strength 60–70% of the opposite side and complete motor recovery in the lower limbs. There was though incomplete recovery of the sensory component.

The patient was ambulatory independently and regained his occupational activities.

## 3. Discussion

Brown-sequard's syndrome features loss of motor power on the side of the hemicord compression and loss of sensation to temperature and pain on the contralateral side. This is based on the anatomical relationship of the spinothalamic (sensory) and the corticospinal (motor) tracts in the spinal cord. The posterior column may or may not be involved depending on the severity and cause of compression [8].

Herniated cervical disc is a rare cause of BSS. We found only 38 cases reported in the English literature in the last eight decades since it was first reported by Stookey in 1928 [6] (Table 1) [2, 6, 8–24]. The incidence of such a combination has been reported to be 2.6% by Jomin et al. [25]. The mean age of presentation was 47 years (range 25 to 73 years) which included 27 males and 11 females. 

One cervical interspace herniation was seen in most of the cases (34 cases, 89.4%) while two-level disc herniation was found in only four cases (10.5%) [13, 16, 22]. There is no reported case of CDH involving more than two-levels causing BSS. 

The level which is most commonly involved is the C5-C6 level in 20 cases (52.6%), while the least being C2-C3 in two cases (5.2%). There were seven cases of C3-C4 (18%), seven of C4-C5 (18%), and six at C6-C7 (15.8%).

The extradural type of CDH was most common, constituting 28 cases (73%). While the intradural CDH has been found to cause severe cord affliction due to direct damage to the cord, extradural discs have a more favourable outcome [2, 23, 24]. The time of presenting also varies, with a mean of 2.4 months (8 days to 18 months) in the intradural cases compared to 5.1 months (8 to 18 months) in the extradural herniations.

 The review of the anatomical location of the disc causing BSS suggests that centrolateral herniation is the most common causes of the syndrome [2–5, 10, 13, 14, 16, 17, 25, 26].

Of the cases reported in detail (Table 1), symptomatic involvement of the spinothalamic tracks was seen in 13 patients (34%), ipsilateral involvement of the corticospinal tracks in nine patients (24%), and axial neck pain or referred extremity pain in 20 patients (53%). Radicular symptoms were seen in only nine patients since the compression involves the cord and not the roots. No patient had posterior column involvement affecting the position and vibration sense, further confirming the partial nature of the lesion.

Of the 38 patients, all underwent surgery. The most commonly adopted approach was the anterior decompression and interbody fusion (ACDF) in 24 patients (63%). Three patients were operated by an anterior corpectomy and fusion (8%), three with anterior discectomy without fusion (8%), and two patients underwent anterior foraminotomy (5%). Posterior surgery in the form of laminectomy or hemilaminectomy was performed mainly in the initially reported literature [10, 14]. Posterior approach to CHD is preferred for a large laterally displaced soft disc, which is seldom found in BSS [27]. The anterior approach is preferred by most surgeons and has been shown to have better results especially for the central and paracentral herniations. It also allows for opening up of the disc space using bone graft [28]. This was also opted in our patient. In our case C5, C6 corpectomy was considered since the C5-C6 and the C6-C7 had migrated behind the corresponding vertebral body caudally. A fusion using a mesh cage and anterior cervical plate was mandatory. This construct provided immediate stability and avoided the problems of pseudoarthrosis and graft failure, if iliac crest of fibular graft alone would have been used [29, 30].

Overall the postoperative recovery after decompression in BSS is satisfactory. Complete recovery was seen in 19 patients (54%), of whom only three had intradural herniation, thus further highlighting the poor prognosis with intradural discs. Pattern of motor recovery and sensory recovery is also variable (Table 1). Our patient also had incomplete motor and sensory recovery at one year followup. Interestingly, it has been found that the MRI hyperintensity seen on the pre-operative images or its persistence in the postoperative scans has no relation with the clinical outcome [24]. Similarly the severity of compression as determined by the neuroradiological investigation and the duration of compression had no bearing on the final outcome [2, 23, 24].

## 4. Conclusion

Brown-sequard's syndrome due to cervical disc herniation remains a rare cause. The diagnosis is further complicated and delayed where classical presentation of BSS is missing. The outcome of this syndrome is favourable provided an early diagnosis is made using an MRI scan. Adequate and prompt surgical decompression and stabilization gives gratifying result.

## Figures and Tables

**Table 1:** Details of the cases reported in the literature.

Reference	Age(Yr)/Sex	Level	Duration	ED	NP+/−LP	MD	SD	Radiation	Surgery	Recovery	MR	SR
Stookey, 1928 [6]	44, M	C3-C4	NR	Yes	+	+		NR	LAM	NR		
	52, M	C5-C6	NR	Yes	+			NR	LAM	NR		
	68, M	C6-C7	NR	Yes	+			NR	LAM	NR		
Düerig and Zdrojewski 1977 [11]	52, M	C5-C6	2 M	No	Thoracic pain			No	LAM		INC	C
Roda et al., 1982 [22]	43, M	C6-C7	1D	No	Thoracic pain			No	LAM		INC	C
Eisenberg et al., 1986 [12]	25, M	C5-C6	4D	No	+			No	LAM		INC	INC
Schneider et al., 1988 [20]	50, F	C5-C6	1D	No	+		+	No	ACD		INC	INC
Sprick et al., 1991 [21]	49, F	C6-C7	10, D	No			+	No	ACDF		INC	INC
Fineli et al., 1992 [13]	28, F	C5-C6	18 M	Yes			+	No	ACDF	No change		
	61, M	C6-C7	8 M	Yes			+	Yes	ACD	CR		
	46, F	C4-C5, C5-C6	18 M	Yes			+	Yes	ACD	CR		
Kobayashi et al., 2003[15]	56, F	C4-C5	5 M	Yes			+	No	ACDF	CR		
Antich et al., 1999 [8]	73, F	C2-C3	6 M	Yes	+			No	ACDF	CR		
Kohno et al., 1999 [17]	33, M	C4-C5	1 M	Yes	NR			NR	ACDF	CR		
	31, M	C5-C6	3 M	Yes	NR			NR	ACDF		INC	INC
	38, M	C5-C6	4 M	Yes	NR			NR	ACDF		INC	INC
	34, M	C3-C4	15 M	Yes	NR			NR	ACDF		INC	INC
	45, F	C4-C5, C5-C6	13 M	Yes	NR			NR	ACDF		C	INC
Börm and Bohnstedt, 2000 [9]	40, M	C5-C6	5 WK	No	+		+	Yes	ACDF	CR		
Clatterbuck et al., 2000 [10]	40, M	C4-C5	5 WK	No	+			Yes	ACDF + LAM		INC	INC
	52, F	C3-C4	2 M	No		+		No	ACDF	CR		
	32, M	C5-C6	9 WK	No		+		No	ACF	CR		
Iwamura et al., 2001 [16]	45, M	C6-C7	15 M	No	+			No	ACF		C	INC
Kobayashi et al., 2003 [15]	64, M	C5-C6	6 M	Yes			+	No	ACDF	CR		
	39, M	C2-C3	1 M	Yes	+			No	ACDF	CR		
Mastronardi and Ruggeri, 2004 [2]	36, M	C5-C6	9 M	Yes	+			Yes	ACDF	CR		
Fujimato et al., 2004 [14]	54, M	C5-C6	3 M	Yes			+	No	LAM		INC	INC
Song et al, 2005, [23]	44, F	C5-C6	6 WK	Yes		+		No	ACDF	CR		
Kim et al, 2005, [23]	56, M	C5-C6	2 M	Yes	+			No	AF	CR		
	46, M	C5-C6	2 WK	Yes	+	+		Yes	AF	CR		
Wang et al, 2006, [23]	44, M	C3-C4	45 D	Yes		+		No	ACDF	CR		
Sathirapanya et al., 2007 [19]	63, M	C5-C6	8 D	Yes	+			Yes	ACDF	CR		
Sayer et al., 2008 [24]	46, M	C3-C4	3 M	Yes	+	+	+	No	ACDF	CR		
Choi et al., 2009 [23]	31, M	C3-C4	4 M	Yes	+			Yes	ACDF	CR		
	66, F	C5-C6, C6-C7	2 M	Yes		+	+	No	ACDF	CR		
	66, M	C5-C6	4 M	Yes	+			No	ACDF		INC	INC
	46, M	C4-C5	2 D	Yes		+	+	No	ACDF		INC	INC
	50, F	C3-C4, C4-C5	3 M	Yes	+		+	Yes	ACDF		INC	INC
Present case	42, M	C4-C5, C5-C6, C6-C7	8 M	Yes	+	+	+	No	ACDF		INC	INC

NR: not reported, D: days, M: months, WK: weeks, ED: extradural herniation, NP: neck pain, LP: limb pain, MD: motor deficit, SD: sensory deficit, LAM: laminectomy, CR: complete recovery, MR: motor recovery, SR: sensory recovery, INC: incomplete recovery.
